# Evaluation of quality and use of health management information system in primary health care units of east Wollega zone, Oromia regional state, Ethiopia:

**DOI:** 10.1186/s12911-020-01148-4

**Published:** 2020-06-12

**Authors:** Mekonen Kebede, Emiru Adeba, Melese Chego

**Affiliations:** 1 Wollega Government Health System, Nekemte, Oromia Ethiopia; 2 Wollega University, Nekemte, Ethiopia; 3 Addis Ababa University, Addis Ababa, Ethiopia

**Keywords:** Health information, Data quality, Primary health care, East Wollega, Ethiopia

## Abstract

**Background:**

Health care practice relies on evidence-based decisions and needs the use of quality health care data. Health management information system (HMIS) is among the core elements of health system building blocks. In our study setting, there is a lack of adequate information on the quality of health information data. This study aimed at exploring the quality of health management information system data in terms of timeliness, completeness, and accuracy. The specific objectives were to evaluate the quality and use of the health management information system in Primary health care units of East Wollega zone, Ethiopia.

**Methods:**

A cross-sectional study was conducted from April to June 2016 on 316 health professionals/health information technicians. The sample was obtained by simple random sampling technique. Qualitative data were obtained from 16 purposefully selected key informants by Focus group discussion (FGD). We observed 50 selected health facilities using an observation checklist. We analyzed quantitative data by SPSS version 20 using descriptive and logistic regression analysis techniques. we applied a thematic analysis approach to analyze qualitative data.

**Results:**

Timeliness of report, registration completeness, report completeness, and data accuracy level of the selected facilities were 70, 78.2, 86, and 48%, respectively. All results are below the expected national standards. Commonly reported reasons for the poor practice of data quality were; poor support of management, lack of accountability for the false report, poor supportive supervision, and lack of separate and responsible unit for health information management.

**Conclusion:**

The Health information management system is poorly coordinated at the primary health units. Accountability should be assured through continuous in-service training, supportive supervision, and concrete feedbacks. Electronic management of health information should be available in primary health care units.

## Background

Since the Alma-Ata declaration in 1978, the World Health Organization (WHO), global partners, and nations emphasized the implementation of Primary health care (PHC). Currently, they are embarking on Universal health coverage (UHC); as a means to attain goal 3 of Sustainable development goals (SDG). The philosophy and principles of PHC promised to assure universal health coverage of essential health services to the large population, especially in rural and remote areas. PHC promotes equity in health service utilization among populations. Despite this fact, there are gaps in evidence-based practice and quality care at PHC level especially in Low and middle-income countries [1–3].

Ethiopian health care facilities expanded rapidly in the past 3 decades. The health care delivery system is organized into three levels, since the Health sector development plan, 2015. Since 2002, the PHCUs serve geographically self-contained health systems at the operational level. Each PHCU is composed of three types of health facilities: Primary hospital, health center, and a health post. Each of the facilities serves 100, 000, 25,000 and 5000 population, respectively [4–6].

A PHC is a decentralized level where much of the original health system data are generated. In Ethiopia, the Health information revolution is one of a key component of health system strategies and plans. However, its quality and use are reported to be weak, particularly in the primary health care facilities [4–6].

Health information management is one of the building blocks of a health system. HMIS quality improves access and quality of service delivery through evidence-based practice. A well-functioning HMIS is an integrated activity of collection, process, analysis, report, and use of health data for decision making in a health system. A well-functioning health system relies not only on the availability of data. Reliable, accurate, and timely information is also vital. Despite the high demand for quality data at PHC levels, evidence shows immense challenges in developing countries, particularly in the primary health care level [7, 8]. Such poor cultures of data use can be improved through the engagement of the health professionals in production, analysis, and interpretation of health information for decision making through performance reviews and workshops [9, 10]. Evidence also shows that, in the health care facilities of developing countries, health professionals are less motivated to use health information at their working units. There is a poor culture of data/information utilization in health facilities and health information is collected mainly for reporting purposes. Studies also stress the need for leadership commitment to the improvement of optimum quality of health information and promoting health information utilization for sound decisions in the health system [11–13].

In developing countries, investments and policies focusing on HMIS are relatively new than the developed country settings. Though the health information demand is increasing, there is poor access to the essential health information for decisions. The collected data are of poor quality and cannot satisfy the need of the users. In developing countries like Ethiopia, it is a pressing time to develop strong and context-based HMIS through innovative approaches [14].

As studies show, HMIS is strongly related to the health system performance and health system strengthening interventions. In resource-limited countries, routine HMIS data with other data types can be used in rigorous evaluation of policy interventions [15]. Furthermore, Integrated HMIS improves the capacity and performance of primary health care facilities and enhances coordination among all levels of health care [16, 17]. Strong HMIS is also a tool for strengthening a health system at facilities through; cost savings, improved access for health resources, and transforming the society for development. Besides, good HMIS promotes the health service utilization pattern of society and increases client satisfaction [18, 19].

Since 2008, the Ethiopian government has a strong stand to strengthen health information; assure 100% coverage of all facilities for routine health care information generation and reporting on national health indicators. Reforms are in place to strengthen the data management system in the health system. In Ethiopia, the health information revolution is one of the four transformation agendas in the health system from 2015 to 2020. As part of the health information revolution, the community health information system (CHIS) was introduced, targeting the local households’ health information data. CHIS is one of the best models and experiences of the Ethiopian health system in health information management. The CHIS is organized in the health posts by the health extension workers (HEWs). The HEWs invest 80% of their time in the community-based outreach activities. However, evidence shows that improving coverage and maintaining the quality and sustainability of such good practices need strong support from external stakeholders [6, 20–22].

As a report from a review of the development of HMIS in Ethiopia, although there are many indicators and information in the health system, health data management, HMIS resources, dissemination of health information, and use of information are poor. The capacity of the health facilities to generate, analyze, interpret, and disseminate the health information across the levels varies, the lower health system level being very weak [23].

A case study focusing on health service delivery achievements in Ethiopia indicated that the decentralization of health care to the population level improved access to health care. However, the local health sector planning, management, and governance capacity is a great challenge. Among the bottlenecks of good management and governance is the fragile HMIS [24].

In a study showing the computerized health information management in India, HMIS is useful for the health workers and program managers. Electronic HMIS saves time and makes data processing and reporting easier. Although the initial cost of computerization is relatively high, there is a good return within 2 years of implementation; if there is a full operation of the system [25].

As reported by measure evaluation’s scale-up project in Southern Ethiopia, there are huge data that are generated daily, monthly, or annually. Such huge data elements are poorly managed and impossible to draw timely and relevant data without the support of an electronic data management system. The report also stressed a need to use the electronic health management information system (eHMIS) as it satisfies the needs of the local system [26].

According to facility-based review on data quality and information use in Ethiopia, standard data collection and reporting tools and operational guidelines are available almost in all facilities. However, data accuracy, report timeliness, and content completeness were 76, 67.7, and 76.7%, respectively, which are lower than the national standards [22]. A similar study in Malawi also indicated the availability of collection and reporting tools in the facilities. However, the study noted infrequent data quality checks and supervisions. Recent Supervision and information use were associated with the availability/accessibility of HMIS data in the facilities [27]. A qualitative study on HMIS data utilization in Pakistan explored that data quality is one of the challenges that hinder the use of HMIS data. The motives behind data management and corruption in the system also affect the quality and use of the data [28]. Another study in Malawi also indicated a lack of qualified staff, low morale of staff, shortage of training, and weak infrastructure were common challenges [29].

On 5 years HMIS quality review in Rwanda, data quality (completeness, consistency) improved from time to time, average monthly report completeness being 98%. Quality improvement was reported to be attributed to strong governance, external supports, rigorous quality improvement approaches, and the performance-based financing system. The use of technologies like web-based data management has improved data quality and information sharing [8].

According to a study in eastern Ethiopia, the overall quality of HMIS data was only 75%. Data quality problems were worst at the lower level of health care facilities like health posts. As indicated in the study, the availability of trained health personnel, supervisory staff, and feedback system were the factors associated with data quality in the facilities [30].

HMIS data quality is a multi-dimensional construct and can be evaluated from different dimensions. According to the WHO data quality guide report card, there is no one and unique definition of data quality across institutions. The dimensions to be assessed and the tools to be used are determined based on the purpose, focus area, and resources at hand [31].

In assuring the quality of the health management information system, top-down approaches have minimum impact. As the majority of the health care burden is to the PHC level, strengthening the lower-level health facilities will have a high impact on assuring evidence-based policy and practice. Although there are recent attempts to strengthen routine HMIS, evidence and good practices are scarce in more decentralized settings like PHCU, particularly in Ethiopia. Thus, the finding will be helpful in HMIS in low and middle-income country settings, as much focus is expected in strengthening primary health care in the coming years to make SDG goal 3 real [1, 5].

### Objectives

The general aim of this study was to evaluate the quality of HMIS data at primary health care units (PHCUs) in East Wollega Zone, Oromia regional state, Ethiopia. The specific objectives of the study included assessing the three dimensions of HMIS data quality (content Completeness, accuracy, and timeliness) and the use of HMIS data in the facilities.

## Methods

### Study setting

East Wollega zone is one of the administrative zones in Oromia regional state, Ethiopia. The zone is bounded in the southwest by Ilubabor Zone, on West by Dhidhesa river/ West Wollega, on North and North West by Benishangul Gumuz, on North-East by Horro Guduru Zone, on East by West Show and on South East by Gibe river/Jimma Zone. Based on the 2007 Census, conducted by the Central statistical agency of Ethiopia (CSA), the zone has a total population of 1,213,503 (Male: 606,379 and Female: 607,124) with an average family size of 4.75. Based on the Census data, only 7.7% of the population is urban and the rest are live in rural (Agriculturalists and pastoralists). The majority of the populations (87.74%) are ethnically Oromo. About 48 % (48.42%) and 37.04% of the population are Evangelical and Orthodox Christians respectively.

In the zone, there are 3 general/referral government hospitals, 17 district health offices, 58 health centers, and 287 health posts. However, the study focused on the PHCU facilities (health centers and health posts).

### Study population

The source population for quantitative data was all health facilities in PHCUs and all-district health offices in the East Wollega zone. The study population was selected health PHCUs facilities and district health offices in the Zone. For qualitative data, purposively selected key informants were from the same health facilities.

### Study design and study period

The institutional-based descriptive cross-sectional study design mixed with qualitative methods (FGD and Observation) was used. The study was conducted from April–June, 2016.

### Sample size determination

The final sample size for quantitative data was calculated using a single finite population proportion formula. The assumptions were, *P* = 32.9% (the proportion of utilization of information and factors affecting data quality(7), degree of precision(d), 5%, Confidence level (95%), the total number of health professionals in the study area (*N* = 2339), non-response rate 5% and adjusting sample size for the small target population (< 10,000).

$${\displaystyle \begin{array}{l}\mathrm{ni}=\frac{{{\mathrm{Z}}^2}_{\alpha /2}\mathrm{P}\left(1-\mathrm{P}\right)}{{\mathrm{d}}^2}=\kern0.75em \frac{1.{98}^2\ast 0.329\ast 0.671}{(0.05)^2}=\mathrm{ni}=346.\\ {}\mathrm{nf}=\frac{\mathrm{ni}}{1+\frac{\mathrm{ni}}{\mathrm{N}}}\kern8em \frac{346}{\left(1+\raisebox{1ex}{$346$}\!\left/ \!\raisebox{-1ex}{$2339$}\right.\right)}=301.\kern2em \mathrm{nf},=301+5\%=316\end{array}}$$

For qualitative data**,** 16 Key informants (Performance monitoring teams and 50 facilities (30 health posts, 15 Health centers, and 5 district health offices) were included in the study.

### Sampling procedure

**Fig. 1:**
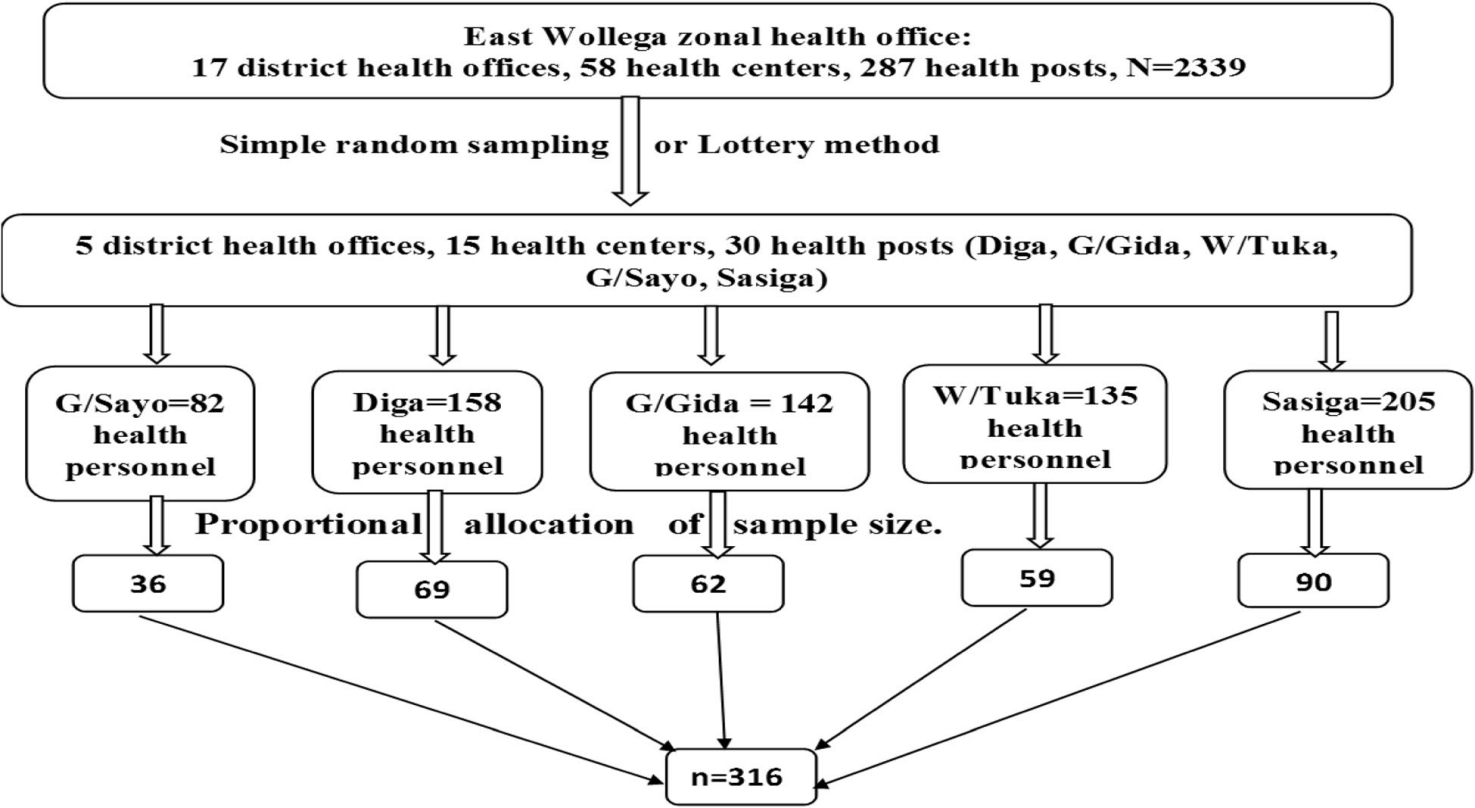
Schematic presentation of sampling procedure, April –June 2016 (*n* = 316)

Thirty (30%) of the total districts (5 PHCUs) were randomly selected and participated in the study. The selected districts were Guto Gidda, Digga, Wayu Tuka, Gobu Sayo, and Sasiga. We included all health centers and 6 selected health posts in the study. The total sample size of health workers was proportionally allocated to each district based on the number of health personnel in the districts (Fig. 1).

### Data collection instrument

To obtain reliable data, we applied the Performance of Routine Information System Management (PRISM) tool. As the study assesses the performance of the health information in primary health care facilities, PRISM is a compressive and appropriate tool. The tool was also used in previous studies [32].

### Data collection and measurement techniques

Quantitative data was collected using face to face interviews with a structured, pre-tested, and standardized questionnaire. The data collectors were 4 BSC level health professionals (Nursing and public health graduates). To measure completeness, accuracy, and timeliness health facility data records and reports were assessed using an observational checklist.

Completeness was assessed from randomly selected 50 cases from each 5 service registration book (OPD Abstract, ANC, EPI, TB, and Delivery) and service delivery and morbidity reports of six consecutive months (October 2015-March, 2016) of each facility. There were 45 health facilities and a total of 225 data registration books were assessed. From the records and reports, completeness for a registration book was measured by calculating the proportion of data cells filled for all cases from the total expected data cells for all cases in a registration book. Cells with absolute zero value, cells having no monthly reportable data elements, and cells corresponding to service that is not given by the health facilities were excluded from the denominator. A minimum value of 85% was considered as complete (Table 3).

For the timeliness of the data report, 12 months (April 2015-March, 2016) service delivery report was observed based on the national reporting period. Health post reports by 21-22th to a health center, a Health center compile, and report to the district health office by 24-26th and District health office compile and report to the zonal health office by 27-2nd month. Timeliness shows the proportion of timely reported data from the expected report during the specified period for the facility or the district. A minimum of 5 out of 6 reports in the specified period was taken as a timely report.

Data accuracy was measured by observing consistency between the reported and recorded data from the service registration book. It was assessed from 12 randomly selected reportable data elements of the 6 months (October–March 2016). The elements of the data assessed for accuracy were, Immunization (Penta 1, Penta 3 and measles), ANC1, Family planning (Implant new), New acceptors of Family Planning, skilled birth attendant, number of adult OPD visits(> = 15 years), percentage of ANC mothers screened for syphilis, PMTCT, and percentage of injectable contraceptive utilization. The verification factor (VF), which was calculated as recounted value divided by the reported value for a data element in each facility, was determined. Data consistency was over-report if VF was below 0.9, acceptable if VF was 0.9–1.1, and under-report if VF was above 1.1.

Qualitative data was collected by FGD. we conducted two FGDs with a total of 16 participants. The number of participants in each FGD was 9 and 7. The FGDs were conducted by MPH graduates using an open-ended FGD guide. The participants of the FGDs were homogenous groups: adult people working in primary health care facilities and the district health officials. They were members of district health offices and Health Center performance monitoring teams. They were selected purposely, as they are responsible for reviewing the health system data and performance in the respective facilities and offices. There were a facilitator and note-taker for each FGD, and data were taken manually with maximum effort. An average of 1 h is used to conduct each FGD.

### Variables

#### Outcome variable

The main outcome variable is data quality (completeness, accuracy, and timeliness). The secondary outcome variable is information use.

### Explanatory variables

- **Technical Factors:** Complexity of reporting form, procedure, HMIS design, software/ application, etc.)
- **Behavioral factors: -**Familiarity with HMIS form, motivation, perception, competence, confidence level for HMIS tasks.
- **HMIS Process:** Data Collection, data processing, data analysis, data presentation, data check, feedback
- **Organizational Factors:** contribution of the management team on HMIS activities, governance of HMIS Activities, planning on HMIS activities, training on data quality and information use, supervision on data quality, promotion of a culture of information use, availability of the resource (human power, budget), and Work Burden.

### Data analysis

Quantitative data were entered, cleaned, and analyzed using SPSS version 20.0. The responses to the Likert scale questions were dichotomized in to, agree, and disagree. The neutral response was considered missing for that specific question. Frequency, mean, and percentages were used to summarize the descriptive results. To identify factors associated with the quality of health information, logistic regression analysis was used. Variables that were significant in binary logistic regression were included in final multiple logistic regression models at a *p*-value of 0.05. The crude and the adjusted odds ratio were used to show the strength of the statistical association.

Qualitative data was analyzed through thematic analysis approach. The raw data that was obtained by note-taking was organized into main themes and sub-themes based on the similarities of the responses. A code was given to each theme and sub-theme. The coded themes and subthemes were sorted by their codes. The seven themes identified were capacity building to HMIS, quality of HMIS data with three subthemes (Completeness, accuracy/consistency, and timeliness) leadership support to HMIS, Utilization of HMIS data, HMIS facilities, Work overload and accountability/ownership to HMIS. Direct quotes of the respondents were also used where it is appropriate. Findings from the qualitative data were used to supplement the quantitative results.

## Result

### Socio-demographic characteristics of the respondents

Among the respondents, 134 (37.0%) were in the age range of 21–30 years with a mean age (SD) of 29.27(±7.41), and 166(52.5%) were female. About 181(57.28%) of the respondents had a salary below the mean salary (3569.15 Ethiopian birrs) ranging from 46 to 275$ per month. About 181(57.3%), and 29(9.2%) were HMIS, and Monitoring & evaluation staff, respectively (Table 1). The profile of FGD participants is indicated in Table 2.

**Table 1:** Distribution of the respondents by their job position, April–June, 2016, (*n* = 316)

Health professional Category	Number (Percent)
Case team coordinator	40 (12.7%)
HMIS Staff/ M& E Team	29 (9.2%)
Technical staff	181 (57.3%)
HEWs (Level III)	49 (15.5%)
HEWs (Level IV)	6 (1.9%)
Head DHO /director of PHCU	11 (3.5%)
**Total**	**316 (100%)**

**Table 2:** Background profile of FGD participants, April 2016 (*n* = 16)

s.no	Key informants	Number		
		Male	Female	Total
**1.**	The medical director of Health center	1	1	2
**2.**	District health manager	2	0	2
**3.**	Health extension worker (HEW)	0	2	2
**4.**	Senior Nurse from the health center	1	1	2
**5.**	District HMIS focal person	1	0	1
**6.**	HMIS focal person of the facilities	1	0	1
**7.**	MCH unit head	0	1	1
**8.**	District health planning officer	1	0	1
**9.**	Health office/clinical officer	2	0	2
**10.**	Laboratory service head, health center	1	0	1
**11.**	Communicable disease control officer of the district	1	0	1

### Capacity building of health professional on HMIS/CHIS in east Wollega zone

**Fig. 2:**
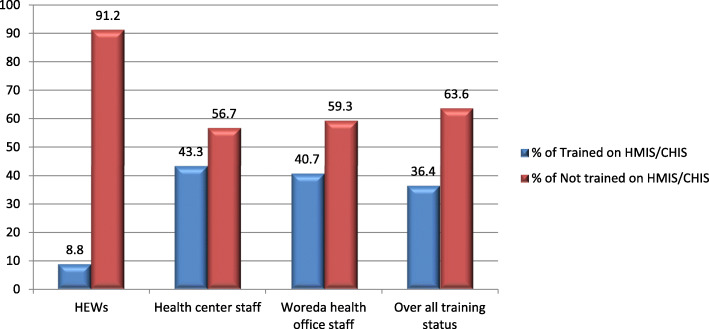
HMIS/CHIS Training status of respondents in the past 6 months, April–June 2016 (*n* = 316)

The study revealed that 201 (63.6%) did not receive basic HMIS/CHIS training in the past 6 months. Only 5(9.1%) of HEWs, 77(43.3%) of the health center, and 33 (40.7%) of district health office professionals received basic training in the past 6 months. The 0verall HMIS training status of the study area was only 115(36.4%) (Fig. 2). The result was also supported by qualitative data. Most of the Focus group discussants agreed that there is no regular scheduled data quality training provided for health professionals, especially at the lower health system level, which is the initial data source. Contrary to this, a 35 years old district health office manager replies, “…*Training is not a matter that is affecting data quality here. The problem is the lack of a good commitment to improving data quality. Most health professionals relate training with the benefit they get from the training…*”. This implies, there is a huge gap in the capacity building of health professionals on health information management in primary health care facilities. The capacity-building activities in primary health care facilities are weak. There is a demand for need-based training to motivate health professionals for quality assurance and information use in primary health care facilities. Training that improves the working environment and the professionals’ skills must be available at the primary health care setting where there is an actual challenge.

### Data quality in terms of completeness

Out of a total of 225 service registration book data, 176 (78.2%) were complete. Out of a total of 200 service delivery and morbidity report data, 172(86%) were complete (Table 3).

**Table 3:** Completeness of registration books and reports of Selected health facilities, April–June, 2016, (*n* = 50)

Type of Health Facility	No of H/Facility	Registration data Completeness (***n*** = 225)		Reported data Completeness (***n*** = 200)	
		Complete	Not Complete	Complete	Not Complete
**Health Post**	30	111 (74%)	39 (26%)	94 (78.3%)	26 (21.7%)
**Health Center**	15	65 (86.7%)	10 (13.3%)	54 (90%)	6 (10%)
**District Health Office**	5	NA	NA	18 (90%)	2 (10%)
**Total**	50	176 (78.2%)	47 (20.8	172 (86%)	28 (14%)

*NA* Not Applicable

The FGD result is also consistent with this finding. It shows that data completeness was not given practical attention. The registration books and reports lack appropriate values. There are zigzag lines and other marks that do not show the important values of the data across the data elements. The response of a 27 years old clinical nurse of a health center strengthens this concern *as “…. Most of the time, I just focus on the assessment of the patient and managing the cases, I think giving attention to recording data may take much time. So, I prefer using different marks like Zigzag to simplify …*”. This implies that the completeness of HMIS data is affected due to the burden of health professionals’ and professionals’ commitment.

### Data quality in terms of timeliness

The study shows that the overall timeliness was only 35(70%). The timeliness of health facility reports to the next respective level of the health system was 21(70%), 10(66.7%), and 4(80%) for Health posts, Health Centers, and district health offices, respectively.

**Fig. 3:**
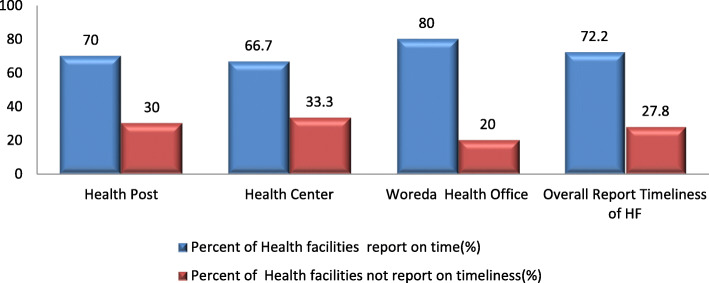
Timeliness of 6 months HMIS report in selected health facilities of east Wollega zone, April–June 2016 (*n* = 50)

The qualitative result also shows that the timeliness of reports is compromised. A late report was observed mostly at a health center level. This is the point where the reports from health posts and health center departments are compiled, entered to eHMIS, generated, and sent to the district health office by soft copy. The completeness of the report is checked at the district level. If errors are identified, the report is returned to the health facilitates for correction. Report editing is impossible at the district health office level (Fig. 3).

### Data quality in terms of accuracy

**Fig. 4:**
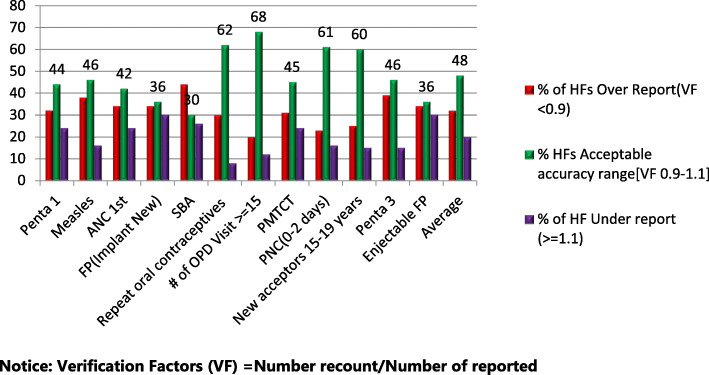
Data accuracy of selected health facilities in East Wollega Zone April–June 2016 (*n* = 45)

Only 24(48%) of the 6 months health facility service delivery report was within the acceptable range (0.9–1.1). About 18(36%) and 8(16%) of health facilities had over-report and under-reports, respectively (Fig. 4). This result was also supported by qualitative responses. A 43 years old man in the FGD claims, *“Data consistency has no attention. I think there is no accountability from the lower to the higher level of the health system. Every individual focuses on reporting purpose rather than keeping data accuracy”*. In the same manner, 24 years old HEW responds as, *“...in most cases, we are concerned about how to satisfy the next health system managers by balancing achievement with a planned target. No clear accountability for false report*s…”. In the presence of low accuracy and high completeness of reports, there is a high probability of false reports sent to the higher facility levels. The weak leadership and supervision support, and high pressure from the leaders to keep timeliness of report justifies high false reports in the Primary health care units.

### Overall data quality

**Fig. 5:**
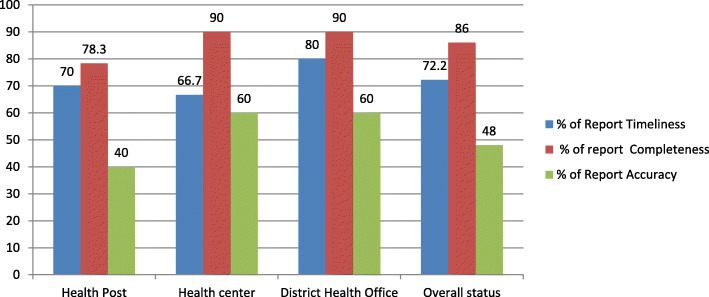
Data quality of selected health facilities in East Wollega Zone, April–June 2016 (*n* = 50)

The study shows that the overall status of data quality in terms of report timeliness, report completeness, and data accuracy was 72.2, 86, and 48%, respectively, which are far less than the national targets. Data quality was slightly improved, looking from a health post to the district health office (Fig. 5).

### HMIS information use

**Fig. 6:**
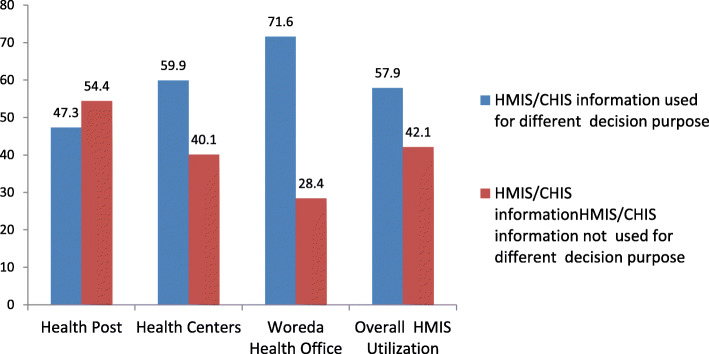
Percentage of professionals that Utilize data in selected health facilities of east Wollega zone, April–June 2016(*n* = 316)

Overall, about 184(57.9%) of health professionals utilized generated HMIS/CHIS information for at least one decision making purpose. This finding was 26(47.3%), 106(59.9%), and 58(71.6%) for Health Posts, Health Centers, and District Health Offices, respectively (Fig. 6).

### Factors associated with HMIS use

As revealed from the multinomial regression analysis, health professionals who get supervisory support were 2.46 times more likely to use HMIS data for various decision-making purposes than their counterparts (AOR: 2.460 CI 95% (1.101,5.493)). In the same way, Health professionals who regularly monitor their performance against the target were 4.070 times more likely to use HMIS/CHIS information for decision-making purposes than those who don’t monitor (AOR: 4.070 CI 95% (1.290, 12.838)). Adjusted for other variables, health professionals who have got a reward for good work on data quality and information use were 2.072 times more likely to use HMIS/CHIS information for various decision purposes than those who haven’t got a reward (AOR: 2.072 CI 95 (1.120,4.294)) (Table 4).

**Table 4:** Factors associated with HMIS/CHIS information used for various decision purposes in district health facilities of East Wollega Zone, April 2016. (*n* = 316)

Variables	HMIS data Information Use	Sig. ***P*** Value	COR	95% CI (COR)	Sig. ***P*** Value	AOR	95% CI (AOR)
**Collecting information is a burden to me**	Agree	0.015	1.151	(1.028,1.288)	0.257	1.483	(0.750,2.932)
	Disagree		1^R^			1^R^	
**I have Confidence in calculating summaries of HMIS**	Agree	0.029	1.309	(1.069,1.604)	0.070	1.972	(0.947,4.106)
	Disagree		1^R^			1^R^	
**Decisions are on worker’s Personal preference**	Agree	0.042	0.814	(0.667,0.992)	0.465	0.729	(0.335,1.586)
	Disagree		1^R^			1^R^	
**There is Supervisory support on Decision**	Agree	0.002	1.396	(1.131,1.724)	0.028	2.460	(1.101,5.493)
	Disagree		1^R^			1^R^	
**Managers discuss openly to resolve problems**	Agree	0.021	0.774	(0.622,0.962)	0.014	0.356	(0.156,0.810)
	Disagree		1^R^			1^R^	
**Managers use HMIS data for monitoring targets**	Agree	0.024	0.734	(0.561,0.960)	0.049	0.456	(0.208,0.997)
	Disagree		1^R^			1^R^	
**Staff monitor their performance against target**	Agree	0.046	1.364	(1.005,1.849)	0.017	4.070	(1.290,12.838
	Disagree		1^R^			1^R^	
**Staff use HMIS for interventions**	Agree	0.006	0.619	(0.440,0.872)	0.129	0.428	(0.143,1.280)
	Disagree		1^R^			1^R^	
**Staff get rewarded for good work on data management**	Agree	0.003	1.192	(1.063,1.337)	0.045	2.072	(1.120,4.294)
	Disagree		1^R^			1^R^	
**No Complexity of HMIS/CHIS System for information users**	`Agree	0.031	0.820	(0.679,0.989)	0.009	0.384	(0.187,0.788)
	Disagree		1^R^			1^R^	

1^R^: Reference Category, Information use referent group was: No (Not used)

## Discussion

This study tried to assess the current status of data quality and information use in the primary health care facilities of the East Wollega zone using the PRISM framework of HMIS/CHIS performance diagnostic tool. Considering job training on HMIS/CHIS data quality and information use, only 38.3% of the respondents reported that they were trained in the past 6 months. Continuous training on data management is important to have a skilled human resource that is confident and motivated to perform an HMIS task.

A similar study conducted in Jimma in 2009 shows that HMIS training was below 50%. Although the health system administrators claim there is optimum training for health professionals on data quality, evidence from the qualitative and quantitative reports indicate low focus towards capacity building on data management. This may create great difficulty in the target of the government to transform districts in terms of health information. Although the health information revolution concept of the government seems timely, it will be ineffective without the behavioral transformation of the health workers, who are engaged in the daily generation of health data. The development of health information quality and use culture has to be a focus at the primary health care level through capacity building tasks [28].

To check the accuracy of the data collected and report at the origin of the data source, the service registration book and copy of monthly HMIS reports should be kept well. According to this study, 66.8% of the respondents document activities and keep records daily. Data accuracy was only 48%, which was far below the Ethiopian national standard of data accuracy of 90%. This finding is also lower than the study done in the Dire Dawa Administrative health facility that shows data accuracy of 75% [29]. This low level of data accuracy may be due to lack of training on the effective use of HMIS/CHIS tools, lack of attention to the management on performance monitoring, and data quality. As evidence from qualitative data shows, this low accuracy and recording level is highly attributed to low motivation, low skill, and work overload of the health professionals at primary health care facilities. Manual and traditional approaches to data management at health facilities made data management activity difficult. This implies a need to develop electronic and digital data management systems in the health facilities if the attempt is to transform data quality in the health system. Presenting guidelines, documents, and registers are merely not enough.

Concerning to timeliness, 72.2% of monthly health facility and District health office service delivery reports were reported as per the Ministry of health schedule. The set time for reporting is within 20th to 22nd days of the month for health post and 24th to 26th for health centers, 27th -2nd day of next month for district health office. A similar study in North Gondar showed only 50% monthly service delivery reports of HMIS were submitted timely [24, 30].

Timeliness of report was high, compared to the study in Jimma, 62% in 2009. This increment may be because the majority of health facilities assigned responsible HMIS professionals as focal persons and familiarity with reporting formats of health personnel improved from time to time [28].

Even though staff commitment and motivation towards good data management and data quality is crucial for assuring data quality, the finding of this result shows that a low level of staff motivation (52.2%). This finding is slightly higher compared to the research done on HMIS data quality and information use at Yekatit 12 hospital in Addis Ababa which was 45.2% [31]. However, this result was lower compared to PRISM based study in SNNPR, Ethiopia, 65% [25]. The reason for low staff motivation may be lower staff incentives.

The strength of the study lies in the attempt to address the peripheral health facilities like health posts and the use of the mixed methodology. However, the study did not include the primary hospitals as the structure is new, and there is no well-established HMIS in such hospitals. The short duration of the study may not rule out the seasonality impact.

As this is a study on the front-line health system structure, the findings will have credible input for HMIS and health system strengthening programs in Ethiopia and similar contexts. The study also implies a need for further descriptive and analytical studies on compressive HMIS, including electronic and community-based health information systems with quantitative and qualitative methods.

## Conclusion

This assessment revealed low data quality in all dimensions, which is below the national standards. Data completeness (86%), report timeliness (72.2%), and data accuracy (48%). Technical training should be given for all health professionals and technical workers on HMIS data management at primary health care units. Leadership and regular supportive supervision should be strengthened. Strengthening the electronic-based HMIS system should be a priority task to improve the dimensions of data quality.

## Data Availability

The data sets during and/or analyzed during the current study available from the corresponding author on reasonable request.

## Abbreviations

HMIS: Health management health information
PHCU: Primary health care units
PRISMA: Performance of routine information system management
FMOH: Federal ministry of health
SNNPE: Southern nation nationalities and peoples of Ethiopia
HSTP: health sector transformation plan
eHMIS: Electronic health management information system
